# Comparative analysis of rhizosphere microbial communities in monoculture and mixed oak–pine forests: structural and functional insights

**DOI:** 10.3389/fmicb.2025.1646535

**Published:** 2025-07-25

**Authors:** Liang Qiao, Zhuizhui Guan, Fangfang Ren, Tianxiao Ma

**Affiliations:** ^1^College of Forestry, Xinyang Agriculture and Forestry University, Xinyang, China; ^2^Xinyang Forestry Technology Workstation, Xinyang, China

**Keywords:** rhizosphere, microbial communities, mixed forests, microbial function, *Pinus massoniana*-*Quercus acutissima*

## Abstract

The ecological significance of rhizosphere microbiomes in forest ecosystems is increasingly recognized. This study provides comparative analysis of microbial communities in *Pinus massoniana*-*Quercus acutissima* mixed forests versus monoculture systems. Mixed stands exhibited superior rhizosphere nutrient conditions and supported more diverse microbial populations, particularly with respect to Proteobacteria, Actinobacteria, and Basidiomycota. Principal component analysis revealed clear separation between rhizosphere and non-rhizosphere soil communities, as well as distinct clustering patterns between mixed and pure forest types. The functional analysis revealed conserved metabolic pathways across forest stands, with bacterial metabolic processes and fungal saprophytic functions representing dominant community roles. Network topology analysis demonstrated enhanced connectivity in mixed forest rhizosphere systems, featuring Proteobacteria, Acidobacteria, and Actinobacteria as bacterial network hubs, while Basidiomycota emerged as central fungal network components. Environmental drivers exhibited differential influences, with bacterial assemblages responding primarily to soil pH, organic carbon content, and phosphorus availability, whereas fungal communities showed stronger associations with organic carbon and potassium levels. These findings collectively demonstrate that mixed-species plantations foster robust microbial networks through microenvironmental regulation, offering valuable insights for sustainable forest management practices.

## 1 Introduction

The rhizosphere, as a critical plant–soil interface, represents the soil microenvironment predominantly shaped by root exudates and microbial activity (Smith, 1976; Han et al., 2023). Characterized by intensive biogeochemical cycling and energy transformation, this dynamic zone serves as a key regulator of nutrient fluxes in forest ecosystems (Kuzyakov and Razavi, 2019). Recent studies have demonstrated that variations in forest vegetation composition—particularly between pure (monoculture) and mixed-species stands—play a critical role in shaping soil microbial community structure and diversity (Gilliam et al., 2023; Gilliam, 2025). Although numerous investigations confirm that mixed-species plantations modify soil microbial resource distribution, existing research predominantly treat soil as a homogeneous entity while neglecting the distinct responses of microbial metabolic activity in rhizospheric versus non-rhizospheric soils to tree species mixing. Substantial evidence indicates that rhizospheric soils exhibit distinct characteristics compared to non-rhizospheric soils, particularly in C/N/P stoichiometry, microbial assemblage structure, and enzymatic activities governing nutrient transformation processes (Gan et al., 2021; Ling et al., 2022; Ma et al., 2023). Understanding the mechanisms underlying mixed-species effects on soil stoichiometric traits (rhizosphere vs. non-rhizosphere) and microbial functional metabolism is essential for deciphering tree diversity-driven biogeochemical processes, particularly in microbial niche differentiation and carbon-nutrient coupling dynamics.

The introduction of multi-species tree mixtures generally enhances soil microbial biomass and diversity while modifying community composition (Pereira et al., 2019; Li et al., 2021). Notably, mixed plantations of *Pinus massoniana* and *Schima superba* demonstrate elevated soil nitrogen mineralization rates, coupled with increased rhizosphere microbial biomass and a higher fungal-to-bacterial ratio (Wu et al., 2022). Comparative studies reveal that mixed poplar (*Populus* spp.) and black locust (*Robinia pseudoacacia*) stands exhibit greater rhizosphere microbial abundance and biochemical activity than monocultures (Hu et al., 2002). Similarly, soil bacterial diversity in monoculture *Cunninghamia lanceolata* plantations is significantly lower than in mixed conifer-broadleaf forests, though fungal diversity displays an inverse trend (Liu et al., 2010). Furthermore, soil fungal communities in pure Pinus stands are dominated by saprophytic taxa, whereas symbiotic fungi become predominant when pines are interplanted with broadleaf species (Wu et al., 2019; Li et al., 2021). These findings suggest that mixed forest management fosters cross-kingdom microbial interactions (e.g., bacteria-fungi and saprotrophic-symbiotic fungal guilds), which drive the reorganization of soil microbial communities into novel structural and functional configurations compared to monocultures (Garau et al., 2019; Guo et al., 2023). Nevertheless, mechanistic understanding of microbial network dynamics in mixed forest soils remains limited. Recent network analyses reveal strengthened positive edge correlations in mixed forest microbial co-occurrence patterns, implying tree species diversity preferentially enhances microbial cooperation over competitive exclusion (Ding et al., 2022).

*P. massoniana*, the predominant timber species and pioneer tree in subtropical China’s silviculture systems, has become extensively naturalized across this ecoregion due to its exceptional growth vigor, superior wood properties, and broad ecological plasticity. Its adaptive traits—including drought tolerance, low soil fertility tolerance, and phenotypic resilience—underpin both large-scale commercial plantations and critical ecosystem services like erosion control and degraded land rehabilitation (Tang et al., 2024; He et al., 2025; Tang et al., 2025). However, silvicultural practices emphasizing monoculture plantations of *P. massoniana*—particularly successive rotation regimes—have precipitated ecological degradation mirroring global monoculture syndromes: biodiversity depletion, constrained nutrient cycling, pathogen susceptibility, chronic decline in stand productivity, and compromised carbon sequestration capacity. These systemic dysfunctions fundamentally constrain multi-objective sustainable management frameworks for masson pine ecosystems (Wen et al., 2019; Li et al., 2014). Ecological restoration trials demonstrate that admixing native broadleaved species (e.g., *Quercus acutissima*) through near-natural silvicultural conversion enhances stand structural complexity, accelerates nutrient turnover, and reinstates carbon sink resilience (Zhao et al., 2016; Zhang et al., 2023).

The introduction of *Q. acutissima* into monoculture *P. massoniana* plantations induces significant alterations in litterfall dynamics, root biomass allocation, and rhizodeposition profiles (Zhang et al., 2023; Zhao et al., 2016). These shifts are hypothesized to drive stoichiometric reorganization in soil matrices and restructure microbial functional guilds across rhizospheric versus bulk soil compartments. This study addresses two critical knowledge gaps: (1) While mixed-species systems are known to enhance soil nutrient availability, their specific mechanisms in modulating microbial functional redundancy and keystone taxa selection remain unresolved; (2) Microbial community assembly drivers between root-influenced versus bulk soil zones under mixed-species regimes requires to be identified. This study investigates the ecological effects of *P. massoniana*-*Q. acutissima* mixed plantations by comparatively analyzing rhizosphere and bulk soils from three distinct forest management systems. Through comprehensive characterization of soil physicochemical properties (pH, nutrient availability) and microbial community profiles, we hypothesize that: (i) interspecific facilitation in mixed stands will enhance soil N/P availability and subsequently shift microbial community structure; (ii) Tree species mixing strengthens microbial network connectivity.

## 2 Materials and methods

### 2.1 Overview of the study area

**Figure 1:**
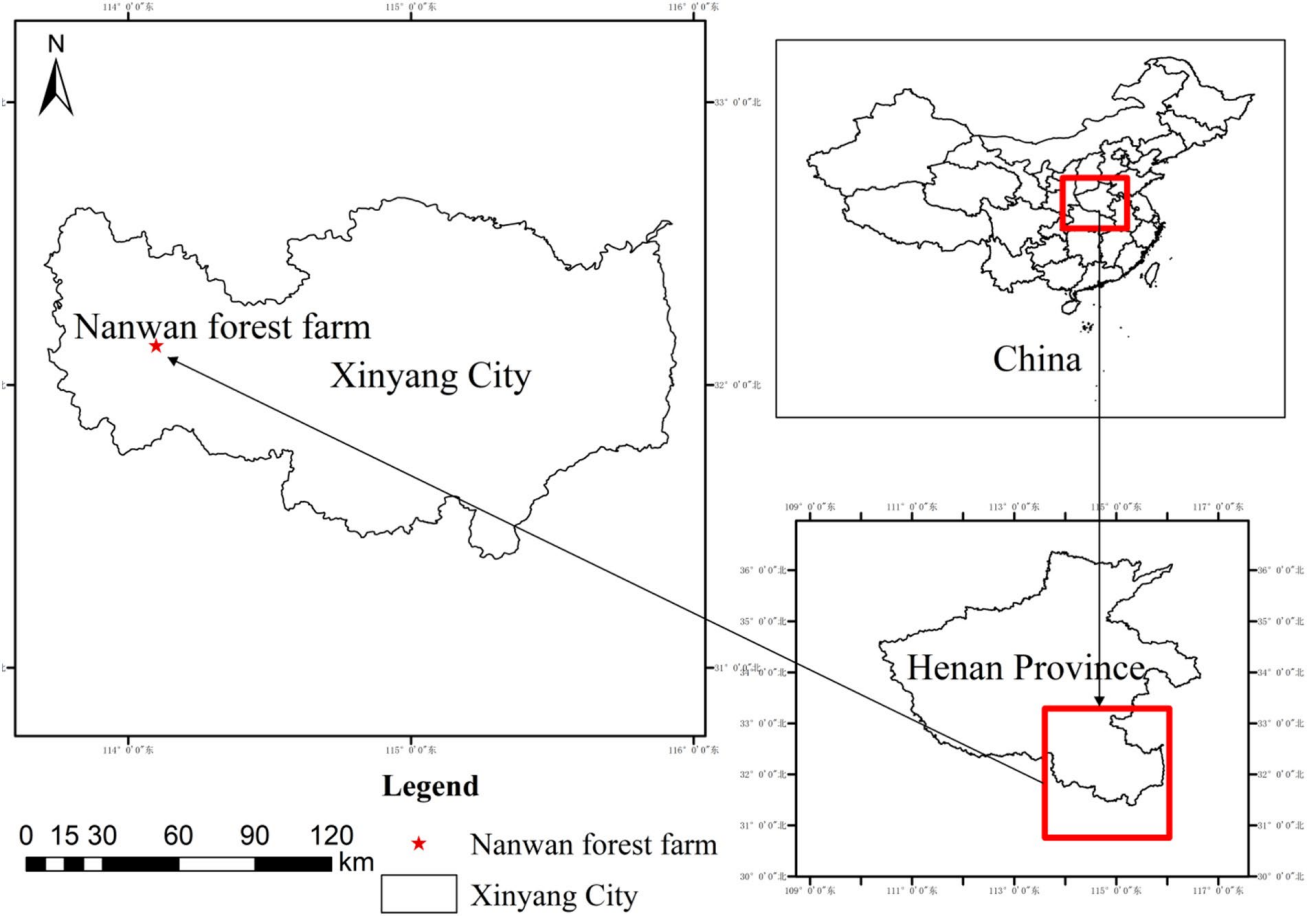
Schematic illustration of sampling locations.

The study was conducted at the Nanwan Experimental Forest Farm in Xinyang City, Henan Province, China (31°49′–32°14′N, 113°45′–114°10′E) (Figure 1). The forest farm spans 8,487 ha, with a forest coverage rate of 98.6% and a total standing timber volume of 876,000 m^3^. Dominant tree species include *Cunninghamia lanceolata*, *Pinus massoniana*, *Quercus acutissima*, and *Liquidambar formosana*. This region has a subtropical continental monsoon climate, featuring four distinct seasons: spring (dry, windy), summer (hot, rainy), autumn (cool), and winter (cold, snowy). Climatic data indicate an average annual temperature of 15.1°C and mean yearly precipitation of 1109.1 mm. The dominant soil type is yellow-brown soil.

### 2.2 Investigation object

The study focused on three forest types: pure *P. massoniana* stands, pure *Q. acutissima* stands, and mixed stands of *P. massoniana* and *Q. acutissima* (hereafter referred to as mixed forests). The *P. massoniana* pure stands are 47 years old, with a stand density of 625 trees·ha^−1^, mean tree height of 19.5 m, and mean diameter at breast height (DBH) of 26.2 cm. The *Q. acutissima* pure stands are 42 years old, with a stand density of 833 trees·ha^−1^, mean height of 13.3 m, and mean DBH of 18.1 cm. In the mixed stands (42 years old, 833 trees·ha^−1^), *P. massoniana* exhibits a mean height of 19.1 m and mean DBH of 24.9 cm, while *Q. acutissima* averages 12.8 m in height and 13.2 cm in DBH.

### 2.3 Soil sample collection

In late July 2024, five randomly selected plots were established in each of the three forest stands, maintaining a minimum spacing of 20 m between plots. Each plot measured 900 m^2^ (30 × 30 m), resulting in a total of 15 sampling plots. From each pure forest plot, five healthy, vigorously growing trees were randomly chosen, and both rhizosphere soil (0–30 cm depth) and non-rhizosphere soil samples were collected for analysis. Rhizosphere soil was collected via the shaking method with maintained sterility (Bai et al., 2024). Two soil types from the same plot were individually mixed, sieved (0.85 mm-sieve), split into two portions, and labeled. Part of the soil samples were maintained at ambient temperature for physical and chemical characterization, whereas the rest were immediately transferred to the lab and frozen at −80°C for microbial analysis. In the mixed forest plot, rhizosphere and non-rhizosphere soil samples were collected from the intermediate zone between *P. massoniana* and *Q. acutissima* (Supplementary Figure S1). Sampling procedures and analytical measurements were consistent with those used in pure stands. Three random sampling points per plot were selected for soil moisture determination via the ring knife method. Post-drying measurements revealed moisture contents of 15.37% (pure *P. massoniana*), 15.34% (pure *Q. acutissima*), and 16.71% (mixed forest), demonstrating slightly higher water retention in mixed stands.

### 2.4 Determination of soil chemical properties

Soil chemical properties were analyzed following the methodology outlined by the literature (Lu, 2002). The measured parameters included soil pH, organic carbon (SOC), total nitrogen (TN), total phosphorus (TP), total potassium (TK), alkali-hydrolyzable nitrogen (AN), available phosphorus (AP), and available potassium (AK). Soil pH was measured potentiometrically. Soil organic matter (SOM) was quantified via the potassium dichromate oxidation-colorimetric method, and SOC content was derived by dividing SOM by the conventional factor of 1.724. TN was determined using the Kjeldahl method, while TP and TK were measured via the molybdenum antimony colorimetric method and hydrofluoric-perchloric acid digestion, respectively. AN was analyzed using the alkaline hydrolysis diffusion method. AP was extracted with hydrochloric acid-ammonium fluoride, and AK was determined via ammonium acetate extraction followed by flame photometry.

### 2.5 Soil microbial determination

#### 2.5.1 DNA extraction, library construction, and metagenomic sequencing

Total genomic DNA was extracted from 30 samples using the E.Z.N.A.® Soil DNA Kit (Omega Bio-tek, Norcross, GA, U.S.) according to manufacturer’s instructions. Concentration and purity of extracted DNA were determined with TBS-380 and NanoDrop2000, respectively. DNA extract quality was checked on 1% agarose gel.

DNA extract was fragmented to an average size of about 400 bp using Covaris M220 (Gene Company Limited, China) for paired-end library construction. Paired-end library was constructed using NEXTflex™ Rapid DNA-Seq (Bioo Scientific, Austin, TX, USA). Adapters containing the full complement of sequencing primer hybridization sites were ligated to the blunt-end of fragments. Paired-end sequencing was performed on Illumina Dnbseq-T7 (Illumina Inc., San Diego, CA, USA) at IGENEBOOK Biotechnology Co., Ltd. (Wuhan, China) using Dnbseq Reagent Kits according to the manufacturer’s instructions.^1^

#### 2.5.2 Sequence quality control and genome assembly

The raw reads from metagenome sequencing were used to generate clean reads by removing adaptor sequences, trimming and removing low-quality reads (reads with N bases, a minimum length threshold of 50 bp and a minimum quality threshold of 20) using the fastp (Chen et al., 2018) (https://github.com/OpenGene/fastp, version 0.20.0). These high-quality reads were then assembled to contigs using MEGAHIT (Li et al., 2015) (parameters: kmer_min = 47, kmer_max = 97, step = 10) (https://github.com/voutcn/megahit, version 1.1.2), which made use of succinct de Bruijn graphs. Contigs with the length being or over 300 bp were selected as the final assembling result.

#### 2.5.3 Gene prediction, taxonomy, and functional annotation

Open reading frames (ORFs) in contigs were identified using MetaGene (Noguchi et al., 2006).^2^ The predicted ORFs with length being or over 100 bp were retrieved and translated into amino acid sequences using the NCBI translation table.^3^

A non-redundant gene catalog was constructed using CD-HIT (Fu et al., 2012) (http://www.bioinformatics.org/cd-hit/, version 4.6.1) with 90% sequence identity and 90% coverage. Reads after quality control were mapped to the non-redundant gene catalog with 95% identity using SOAPaligner (Li et al., 2008) (http://soap.genomics.org.cn/, version 2.21), and gene abundance in each sample were evaluated.

Representative sequences of non-redundant gene catalog were annotated based on the NCBI NR database using blastp as implemented in DIAMOND v0.9.19 with e-value cutoff of 1e^−5^ using Diamond (http://www.diamondsearch.org/index.php, version 0.8.35) for taxonomic annotations (Buchfink et al., 2015). Cluster of orthologous groups of proteins (COG) annotation for the representative sequences were performed using Diamond (http://www.diamondsearch.org/index.php, version 0.8.35) against eggNOG database (version 4.5.1) with an e-value cutoff of 1e^−5^ (Buchfink et al., 2015). The KEGG annotation was conducted using Diamond (http://www.diamondsearch.org/index.php, version 0.8.35) against the Kyoto Encyclopedia of Genes and Genomes database (http://www.genome.jp/keeg/, version 94.2) with an e-value cutoff of 1e^−5^ (Buchfink et al., 2015).

### 2.6 Statistical analysis

Multiple comparisons of soil physicochemical properties, microbial relative abundance, and alpha diversity indices were conducted using two-way analysis of variance (ANOVA) and Tukey’s HSD test (*p* < 0.05). Variations in soil microbial community composition among treatments were evaluated through principal component analysis (PCA) (Vázquez-Baeza et al., 2013). The associations between microbial communities and soil environmental factors were determined via redundancy analysis (RDA) (Bokulich et al., 2018). Microbial co-occurrence patterns were investigated using network analysis. Functional predictions for soil bacteria and fungi were performed using PICRUSt2 and FUNGuild, respectively (Nguyen et al., 2016; Langille et al., 2013).

## 3 Results

### 3.1 Soil indicators

**Figure 2:**
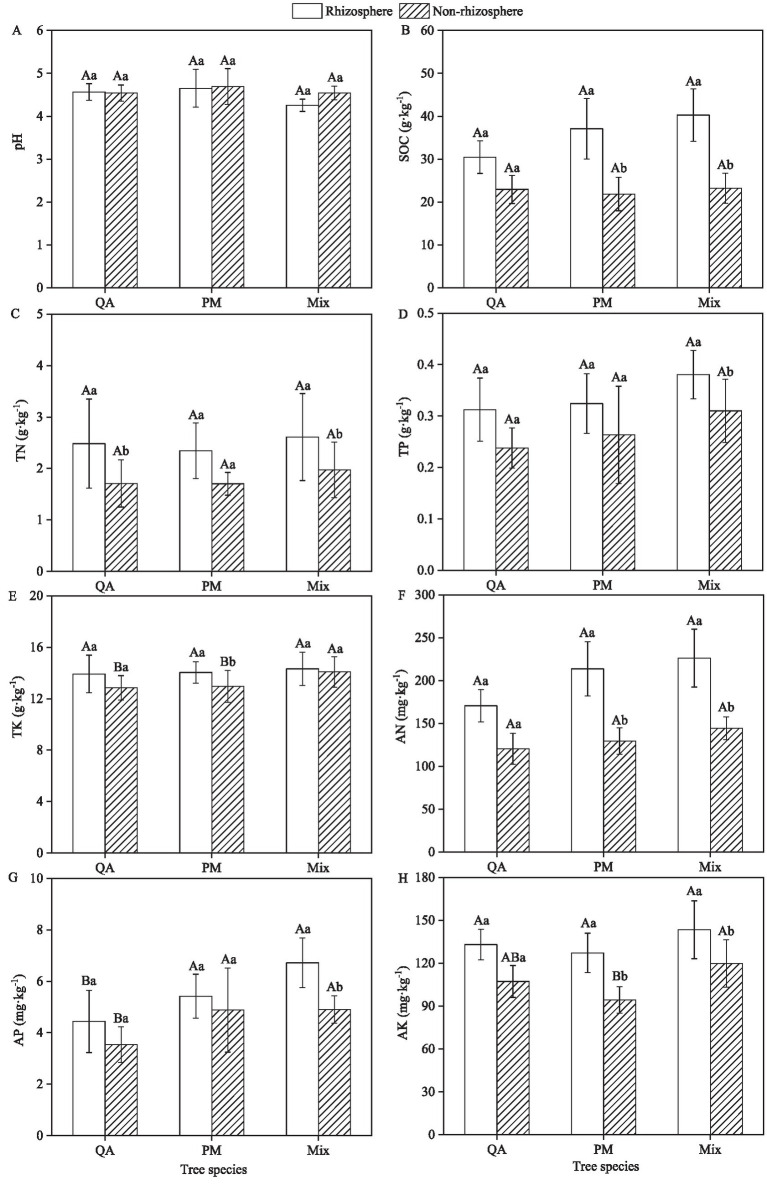
Comparison of soil indicators among different stand types. **(A)** pH; **(B)** SOC; **(C)** TN; **(D)** TP; **(E)** TK; **(F)** AN; **(G)** AP; and **(H)** AK. SOC, Soil organic carbon; TN, Total nitrogen; TP, Total phosphorus; TK, Total potassium; AN, Available nitrogen; AP, Available phosphorus, and AK, Available potassium. Values were means ± SDs, *n* = 5. Different capital letters indicated significant differences among tree species (*p* < 0.05), while different lowercase letters indicated significant differences among soil types (*p* < 0.05). QA, *Q. acutissima*; PM, *P. massoniana*; and Mix, mixed forest of *Q. acutissima* and *P. massoniana*.

Among all measured soil parameters (excluding pH), the rhizosphere soil exhibited higher values compared to the non-rhizosphere soil (Figure 2). Within the same soil type, mixed forests demonstrate significantly greater TP, AP, and AN contents compared to pure stands—with pure PM forests ranking intermediate and pure QA forests displaying the lowest values (Figures 2D,F,G). Mixed forests demonstrated greater SOC, TN, TK, and AK concentrations than both single-species forests (Figures 2B,C,E,H). Compared to pure QA and PM stands, the mixed forest showed significant increases in rhizosphere soil nutrients—32.6 and 5.8% for AN, and 51.4 and 24.0% for AP, respectively (Figures 2F,G).

### 3.2 Composition of soil microbial community

**Figure 3:**
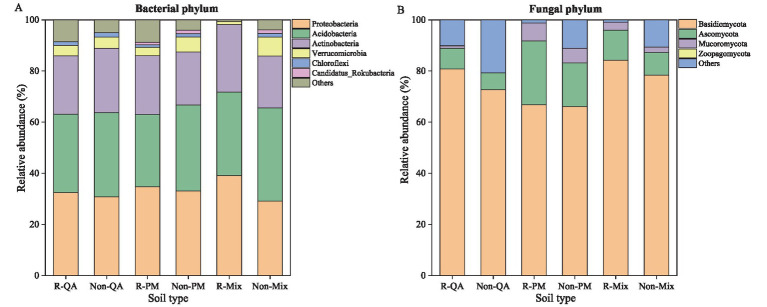
Comparison of the relative abundance of microbial community composition in different soil types. The abundance ≥ 1% in the figure was displayed, while the abundance < 1% was classified as others. **(A)** Bacterial phylum; and **(B)** Fungal phylum. R-QA, rhizosphere soil of *Q. acutissima*; Non-QA, non-rhizosphere soil of *Q. acutissima*; R-PM, rhizosphere soil of *P. massoniana*; Non-PM, non-rhizosphere soil of *P. massoniana*; R-Mix, rhizosphere soil in a *Q. acutissima* and *P. massoniana* mixed forest; and Non-Mix, non-rhizosphere soil in a *Q. acutissima* and *P. massoniana* mixed forest.

**Figure 4:**
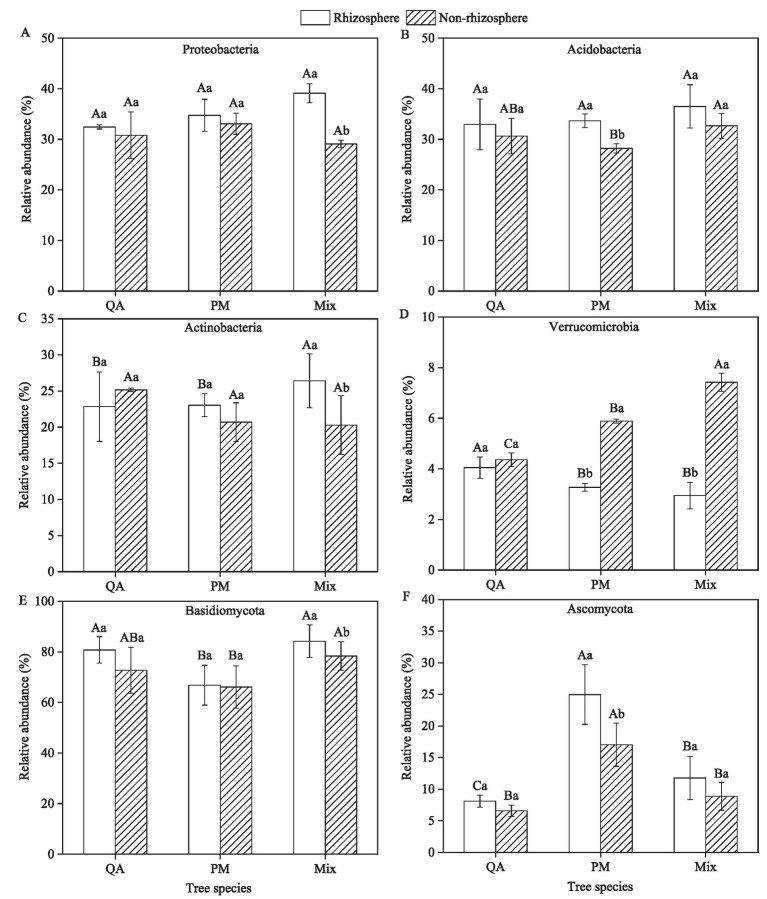
Comparison of rhizosphere differences in relative abundance of soil microorganisms among tree species. **(A)** Proteobacteria; **(B)** Acidobacteria; **(C)** Actinobacteria; **(D)** Verrucomicrobia; **(E)** Basidiomycota; and **(F)** Ascomycota. Values were means ± SDs, *n* = 5. Different capital letters indicated significant differences among tree species (*p* < 0.05), while different lowercase letters indicated significant differences among soil types (*p* < 0.05). QA, *Q. acutissima*; PM, *P. massoniana*; and Mix, mixed forest of *Q. acutissima* and *P. massoniana*.

The predominant bacterial phyla in the soil were Proteobacteria (29.1–39.1%) and Acidobacteria (28.2–36.5%), whereas the fungal community was dominated by Basidiomycota (66.1–84.2%), with Ascomycota (6.6–25.0%) as a secondary major group (Figure 3). Microbial populations in rhizosphere soil showed a greater relative abundance of Proteobacteria, Acidobacteria, Actinobacteria, Basidiomycota, and Ascomycota than those in non-rhizosphere soil (Figures 4A–C,E,F). Conversely, Verrucomicrobia displayed an opposite trend in their distribution between the two soil types (Figure 4D). The rhizosphere soil of mixed forests showed a greater relative abundance of Proteobacteria, Acidobacteria, Actinobacteria, and Basidiomycota than that of the two pure forest types (Figures 4A–C,E). Notably, Proteobacteria abundance increased by 20.5% (vs. QA) and 12.6% (vs. PM), while Actinobacteria increased by 15.6% (vs. QA) and 14.6% (vs. PM) in mixed forests (Figures 4A,C).

### 3.3 Community structure of soil microbes

**Figure 5:**
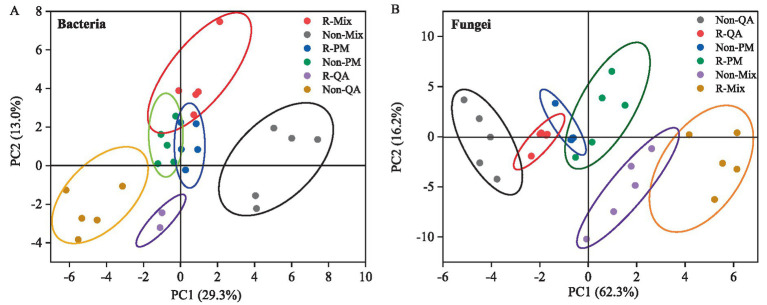
Principal component analysis (PCA) of soil microbial community structure (*β*-diversity). **(A)** Bacteria and **(B)** Fungi. R-QA, rhizosphere soil of *Q. acutissima*; Non-QA, non-rhizosphere soil of *Q. acutissima*; R-PM, rhizosphere soil of *P. massoniana*; Non-PM, non-rhizosphere soil of *P. massoniana*; R-Mix, rhizosphere soil in a *Q. acutissima* and *P. massoniana* mixed forest; and Non-Mix, non-rhizosphere soil in a *Q. acutissima* and *P. massoniana* mixed forest.

In the bacterial community structure, the first two principal components (PC1 and PC2) explained 29.3 and 13.0% of the total variation, respectively (Figure 5A). For fungal communities, PC1 captured a substantially higher proportion (62.3%), followed by PC2 (16.2%) (Figure 5B). The bacterial and fungal community structures exhibited significant differences between rhizosphere and non-rhizosphere soils across all tree species. Additionally, distinct variations in microbial community composition were observed between mixed forests and pure forests, regardless of soil type (Figures 5A,B).

### 3.4 Diversity of soil microbes

**Figure 6:**
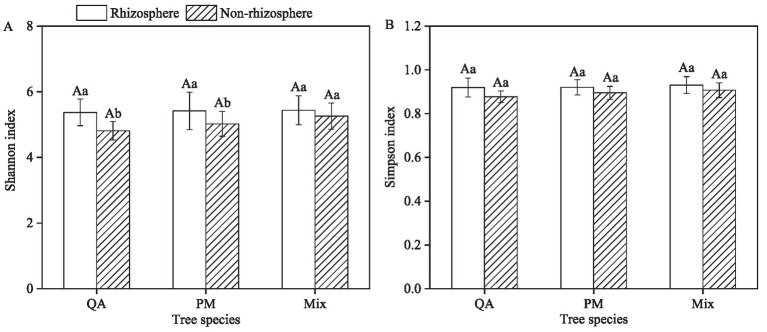
Comparison of rhizosphere differences in soil microbial diversity among tree species (*α*- Diversity). **(A)** Shannon index and **(B)** Simpson index. Values were means ± SDs, *n* = 5. Different capital letters indicated significant differences among tree species (*p* < 0.05), while different lowercase letters indicated significant differences among soil types (*p* < 0.05). QA, *Q. acutissima*; PM, *P. massoniana*; and Mix, mixed forest of *Q. acutissima* and *P. massoniana*.

The Shannon and Simpson index of rhizosphere soil microbes were significantly higher than those in non-rhizosphere soil (Figures 6A,B). The Shannon index of rhizosphere soil microbes exhibited increases of 11.6% (QA), 7.9% (PM), and 3.3% (Mix) compared to non-rhizosphere soil (Figure 6A). Similarly, the Simpson index showed respective increases of 4.9% (QA), 2.8% (PM), and 2.6% (Mix) (Figure 6B). However, the Shannon and Simpson index of soil microbial communities in mixed forests showed no significant differences compared to those in the two pure forests (*p* > 0.05) (Figures 6A,B).

### 3.5 Linkages between soil microbes and soil environmental factors

**Figure 7:**
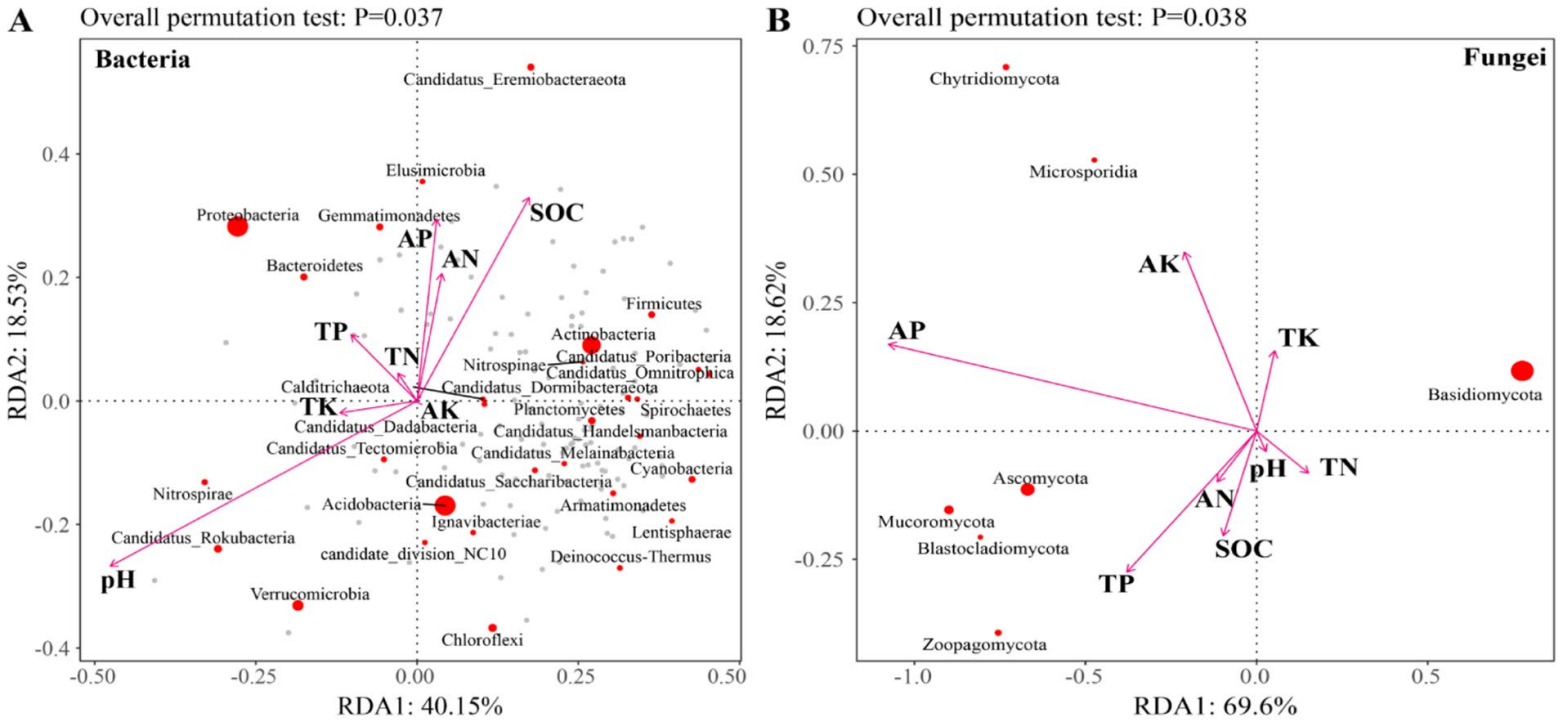
RDA analysis of the relationship between soil microbial community and soil properties. **(A)** Bacteria and **(B)** Fungi. SOC, Soil organic carbon; TN, Total nitrogen; TP, Total phosphorus; TK, Total potassium; AN, Available nitrogen; AP, Available phosphorus; and AK, Available potassium.

**Figure 8:**
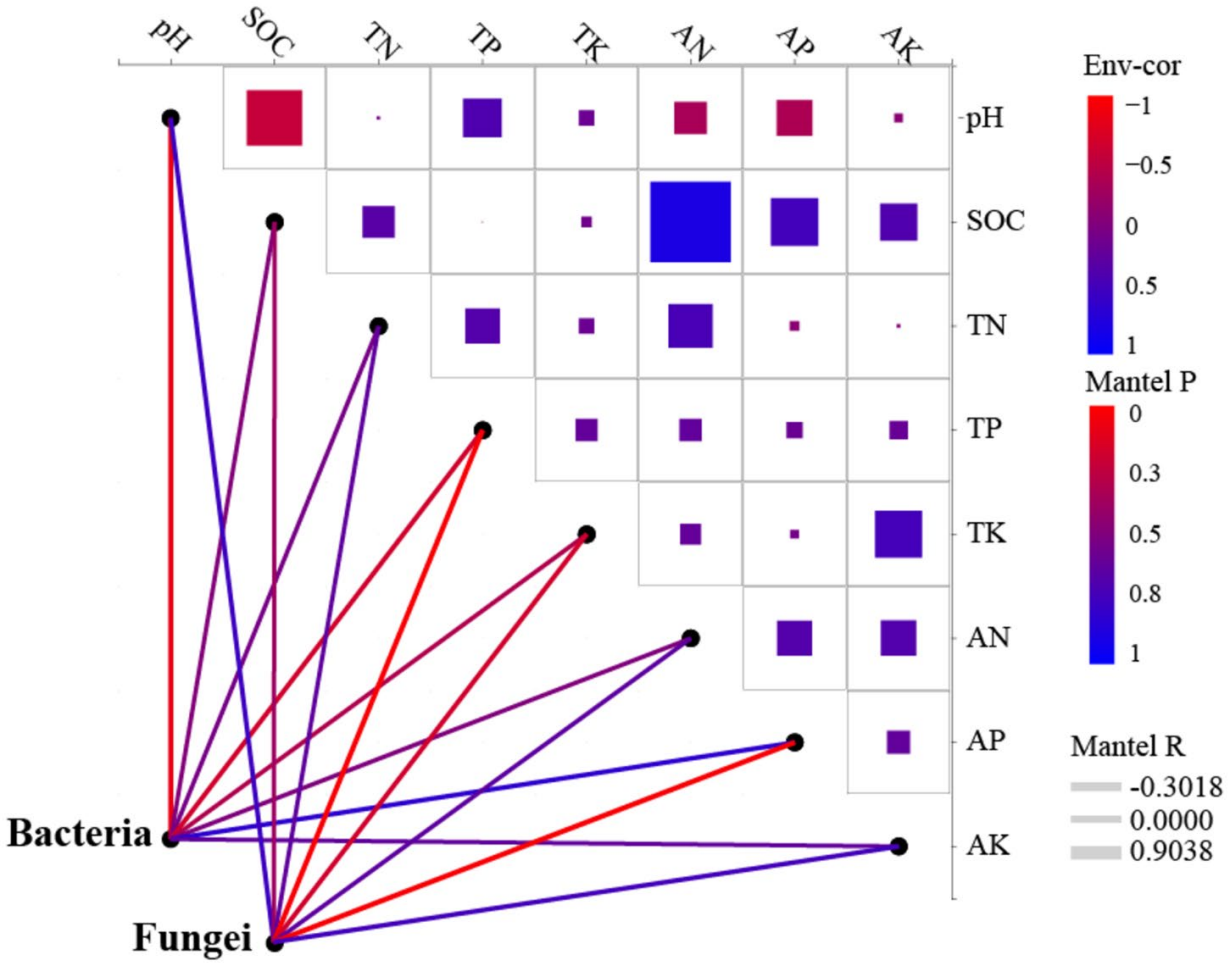
Dynamic network analysis of soil microbial communities. SOC, Soil organic carbon; TN, Total nitrogen; TP, Total phosphorus; TK, Total potassium; AN, Available nitrogen; AP, Available phosphorus; and AK, Available potassium.

Redundancy analysis (RDA) revealed that the first two axes (RDA1 and RDA2) explained 40.15 and 18.53% of the total variance in the bacterial community, respectively (Figure 7A). For the fungal community, RDA1 accounted for 69.60%, while RDA2 contributed 18.62% of the explained variation (Figure 7B). The abundance of bacterial communities was significantly influenced by pH, SOC, TP, AN, and AP, whereas fungal community abundance was primarily affected by SOC, TP, AK, and AP (Figures 7, 8). There was a significant negative correlation between pH and SOC. In contrast, SOC was significantly positively correlated with both AN and AP. Moreover, TK and AK displayed a significant positive correlation (Figure 8).

### 3.6 Network analysis of soil microbes

**Figure 9:**
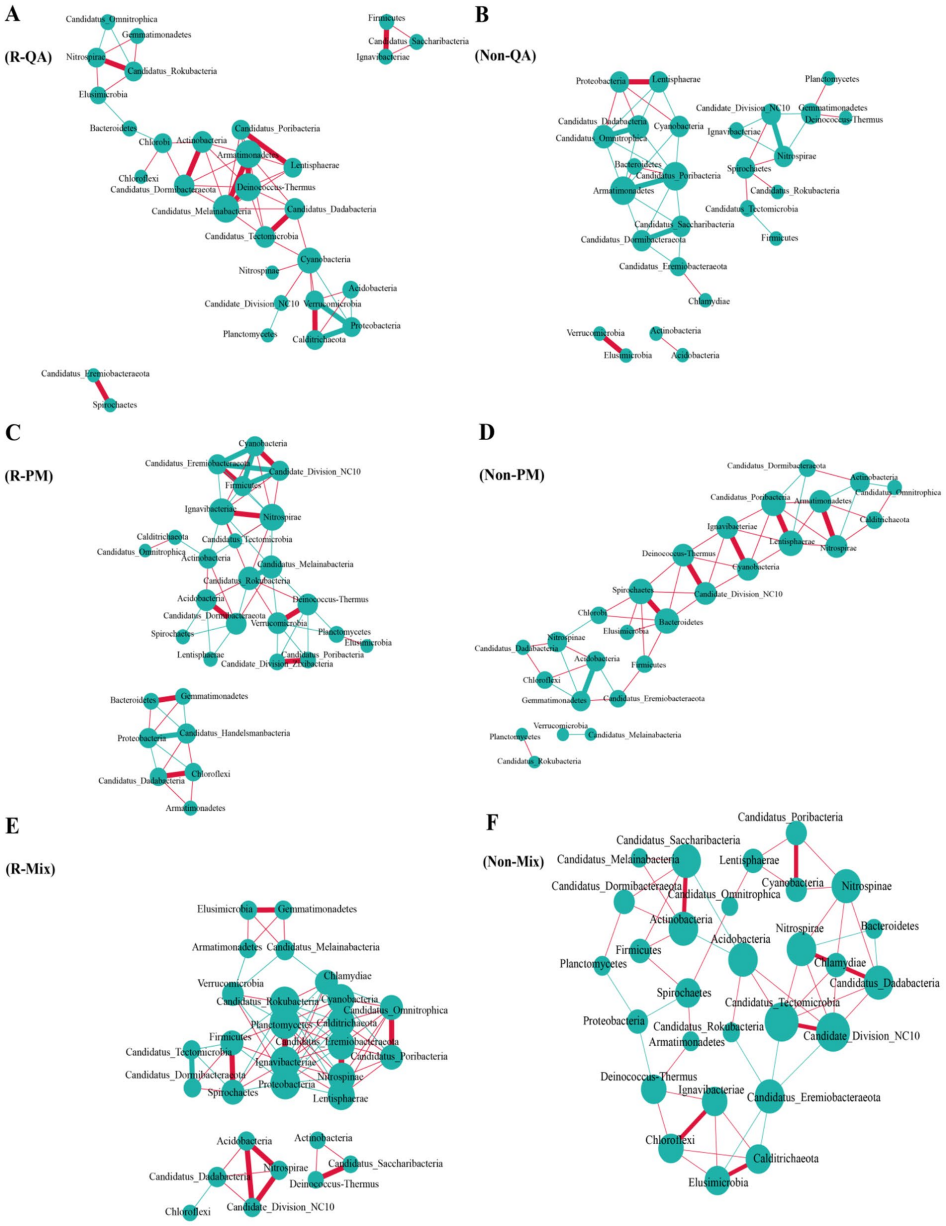
Network analysis of soil bacterial communities. In the visualization, larger circles indicated higher nodes, and thicker lines signified stronger correlations. Positive relationships were highlighted in red, while negative associations were marked in green. **(A)** R-QA; **(B)** Non-QA; **(C)** R-PM; **(D)** Non-PM; **(E)** R-Mix; and **(F)** Non-Mix. R-QA, rhizosphere soil of *Q. acutissima*; Non-QA, non-rhizosphere soil of *Q. acutissima*; R-PM, rhizosphere soil of *P. massoniana*; Non-PM, non-rhizosphere soil of *P. massoniana*; R-Mix, rhizosphere soil in a *Q. acutissima* and *P. massoniana* mixed forest; and Non-Mix, non-rhizosphere soil in a *Q. acutissima* and *P. massoniana* mixed forest.

**Figure 10:**
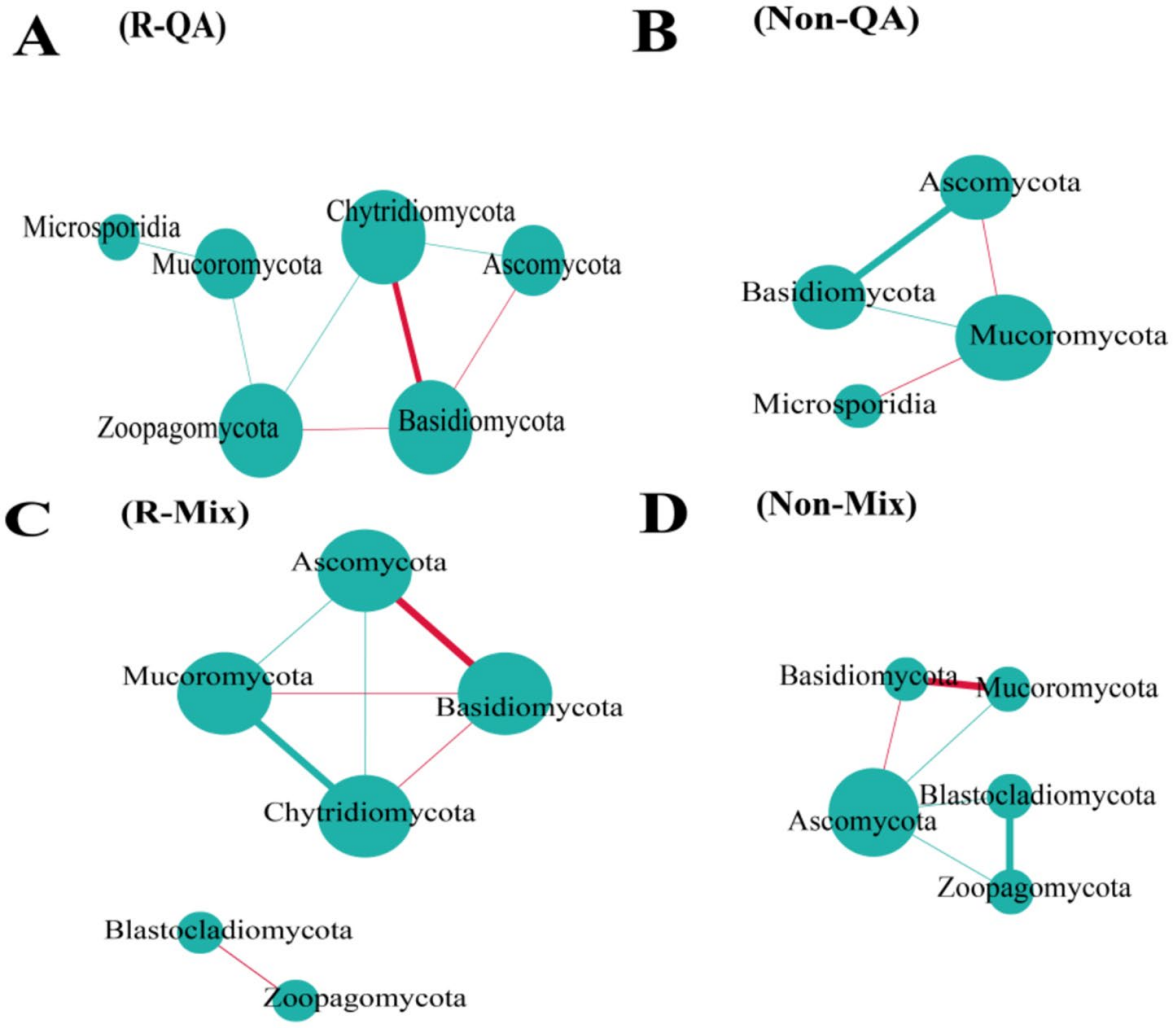
Network analysis of soil fungal communities. In the visualization, larger circles indicated higher nodes, and thicker lines signified stronger correlations. Positive relationships were highlighted in red, while negative associations were marked in green. **(A)** R-QA; **(B)** Non-QA; **(C)** R-Mix; and **(D)** Non-Mix. R-QA, rhizosphere soil of *Q. acutissima*; Non-QA, non-rhizosphere soil of *Q. acutissima*; R-Mix, rhizosphere soil in a *Q. acutissima* and *P. massoniana* mixed forest; and Non-Mix, non-rhizosphere soil in a *Q. acutissima* and *P. massoniana* mixed forest. Fungal communities in PM soil exhibited no significant interactions; thus, they were omitted from this figure. PM, *P. massoniana*.

The rhizosphere soil exhibited a higher number of bacterial connections compared to non-rhizosphere soil, with increases of 38.5% (QA), 28.6% (PM), and 71.2% (Mix), respectively (Figure 9). Across both soil types, mixed forests demonstrated greater bacterial network connectivity than pure forests, while PM forests maintained a higher number of connections than QA forests (Figure 9). Among all soil types, the predominant bacterial phyla—Proteobacteria, Acidobacteria, and Actinobacteria—serve as keystone taxa (Supplementary Table S1). Fungal network connectivity was significantly higher in rhizosphere soil compared to non-rhizosphere soil (Figure 10). However, the difference in fungal connectivity between Mix and pure QA stands was marginal. The dominant fungal phyla across all soil types included Basidiomycota, Ascomycota, and Mucoromycota (Supplementary Table S2).

### 3.7 Functional prediction of soil microbes

**Figure 11:**
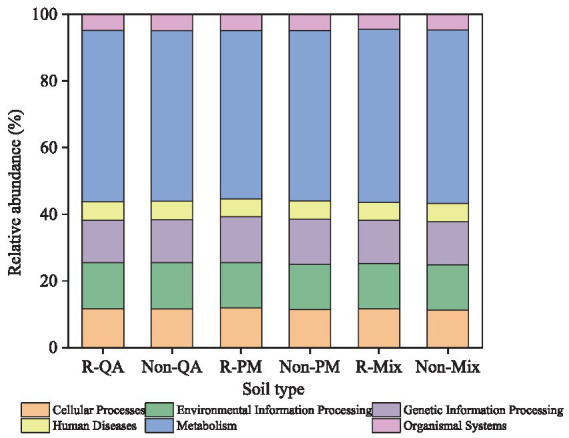
Primary functional analysis of soil bacteria. R-QA, rhizosphere soil of *Q. acutissima*; Non-QA, non-rhizosphere soil of *Q. acutissima*; R-PM, rhizosphere soil of *P. massoniana*; Non-PM, non-rhizosphere soil of *P. massoniana*; R-Mix, rhizosphere soil in a *Q. acutissima* and *P. massoniana* mixed forest; and Non-Mix, non-rhizosphere soil in a *Q. acutissima* and *P. massoniana* mixed forest.

**Figure 12:**
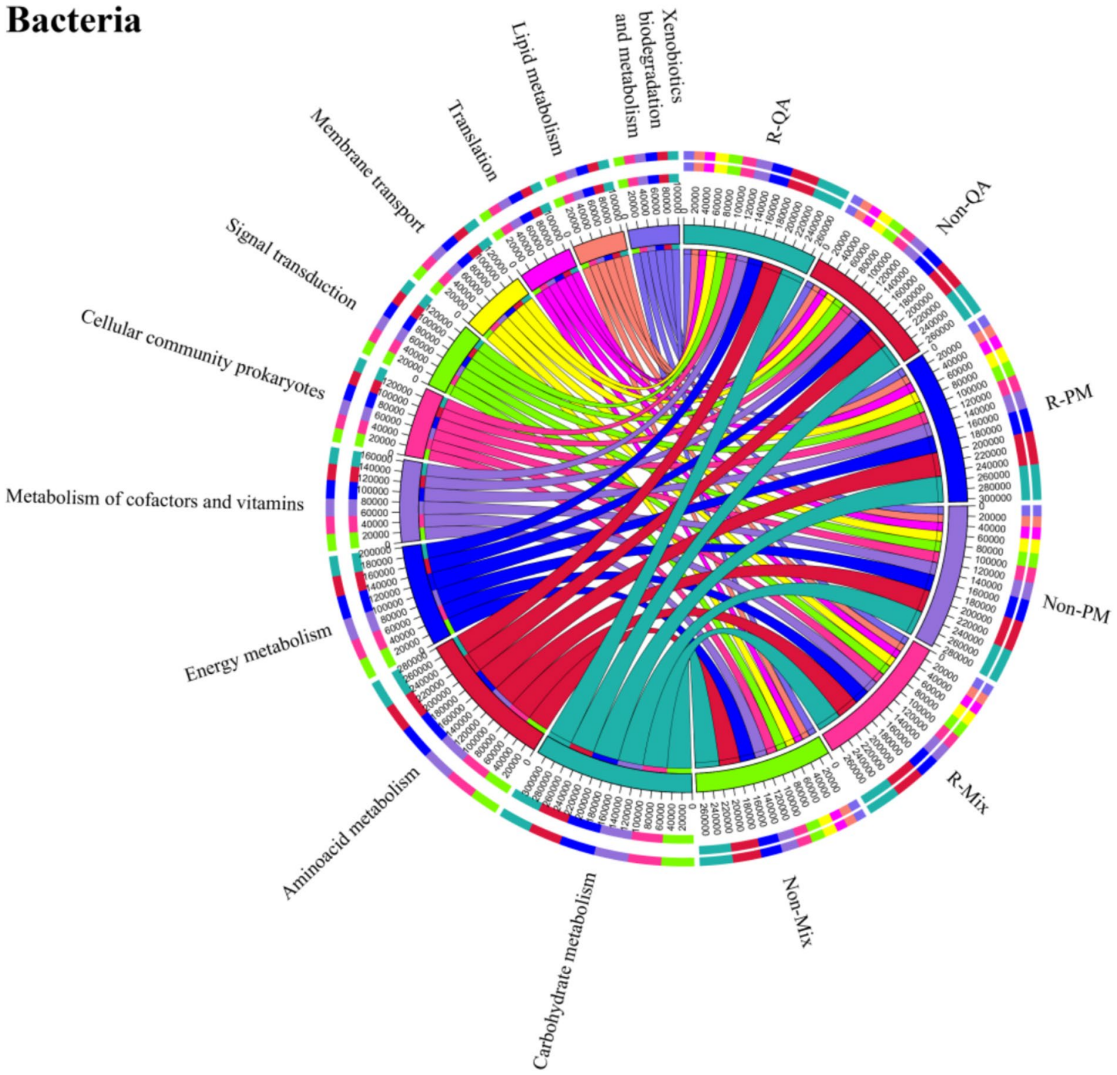
Secondary functional analysis of soil bacteria. Line thickness corresponds to higher functional abundance. R-QA, rhizosphere soil of *Q. acutissima*; Non-QA, non-rhizosphere soil of *Q. acutissima*; R-PM, rhizosphere soil of *P. massoniana*; Non-PM, non-rhizosphere soil of *P. massoniana*; R-Mix, rhizosphere soil in a *Q. acutissima* and *P. massoniana* mixed forest; and Non-Mix, non-rhizosphere soil in a *Q. acutissima* and *P. massoniana* mixed forest.

The core functional roles of soil bacteria encompassed cellular processes, environmental information processing, genetic information processing, human diseases, metabolism, and organismal systems. Among these, metabolism dominated, representing 50.5–52.1% of the total functional abundance (Figure 11). Notably, minimal functional variation was observed across different tree species and soil types (Figure 11). The secondary functions of soil bacteria primarily encompassed xenobiotics biodegradation, lipid metabolism, translation, membrane transport, signal transduction, prokaryotic cellular communities, cofactor and vitamin metabolism, energy metabolism, aminoacid metabolism, and carbohydrate metabolism (Figure 12). Among these, aminoacid metabolism and carbohydrate metabolism represented the dominant secondary functions. Notably, variations in secondary functional profiles across different tree species and soil types were minimal (Figure 12).

**Figure 13:**
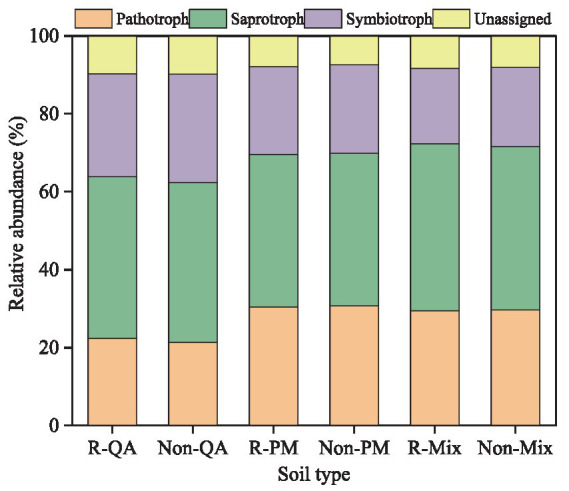
Functional analysis of soil fungi. R-QA, rhizosphere soil of *Q. acutissima*; Non-QA, non-rhizosphere soil of *Q. acutissima*; R-PM, rhizosphere soil of *P. massoniana*; Non-PM, non-rhizosphere soil of *P. massoniana*; R-Mix, rhizosphere soil in a *Q. acutissima* and *P. massoniana* mixed forest; and Non-Mix, non-rhizosphere soil in a *Q. acutissima* and *P. massoniana* mixed forest.

Soil fungi were primarily classified into three functional groups: pathotrophs, saprotrophs, and symbiotrophs. Saprotrophs were the most abundant, comprising 39.1–42.8% of the fungal community, followed by symbiotrophs (19.4–27.8%). Notably, variations in fungal functional composition between different tree species and soil types were minimal (Figure 13).

## 4 Discussion

### 4.1 Changes in soil nutrients

Soil carbon (C), nitrogen (N), and phosphorus (P) constitute fundamental nutrient elements that govern plant growth and mediate biogeochemical cycling at the plant–soil-microbe interface (Lu et al., 2021). The rhizosphere, as a critical zone of plant-environment interaction, exhibits distinct physicochemical properties from bulk soil through pronounced rhizosphere effects (Thacker and Quideau, 2021). Our findings demonstrated a reduction in rhizosphere pH relative to non-rhizosphere soil in mixed forests, aligning with observations by the literature of Xu et al. (2021). This acidification phenomenon primarily stemed from two mechanisms: (1) proton excretion by rhizosphere microbiota during metabolic activity, and (2) release of low-molecular-weight organic acids (e.g., citric, malic acids) through root exudation processes (Yuan et al., 2020). Our findings demonstrated that the mixed plantation of *P. massoniana* (coniferous) and *Q. acutissima* (broad-leaved) significantly enhanced soil nutrient pools (e.g., SOC, TN, and TP) compared to monocultures. These results aligned with the findings of Bai (2023) but contrasted with the report of Huang et al. (2025) on *C. lanceolata*-*Castanopsis fissa* mixed forests. The discrepancy may stem from stand developmental stages: Huang’s study examined 10-year-old Chinese fir during peak growth phases, where high nutrient demand coupled with reduced litter input likely created temporary nutrient depletion (Li et al., 2023). In our system, the substantial litter accumulation and accelerated decomposition rates in *P. massoniana*-*Q. acutissima* stands appeared to drive continuous nutrient replenishment through microbial-mediated mineralization processes.

### 4.2 Microbial community and composition

Recent studies demonstrated that Acidobacteria, a ubiquitous phylum in soil bacterial communities, exhibited substantial variations in relative abundance across different ecosystems (Huber et al., 2016). Members of this phylum possessed remarkable metabolic versatility, particularly in degrading recalcitrant carbohydrates through flexible carbon utilization pathways, thereby playing a pivotal role in soil organic matter cycling (Kalam et al., 2020). Notably, Acidobacteria demonstrated exceptional ecological competence in oligotrophic environments, as evidenced by their predominance in nutrient-poor soils through efficient resource acquisition strategies (Zelenev et al., 2005; Dennis et al., 2010). Our results demonstrated significantly higher relative abundances of Acidobacteria and Proteobacteria in mixed forest rhizospheres compared to pure stands. This enrichment likely reflected two synergistic mechanisms: (1) Enhanced acidobacterial populations promoting organic matter mineralization, thereby elevating nutrient availability (Bai, 2023); and (2) Plant-mediated selection through root exudation, which shaped distinct rhizosphere microbiomes (Garau et al., 2019). Notably, the copiotrophic Proteobacteria showed particularly strong rhizosphere enrichment, consistent with their known capacity for rapid utilization of labile root-derived carbon (Philippot et al., 2013). These findings suggested mixed-species planting created a positive feedback loop where diversified exudate profiles stimulate nutrient-cycling bacteria that in turn supported plant growth.

Our results demonstrated a significant enrichment of Basidiomycota in mixed forest soils compared to pure stands. This fungal predominance correlated strongly with elevated lignin content and acid-unhydrolyzable residue concentrations. As key decomposers of recalcitrant organic matter, Basidiomycota secreted a diverse array of carbohydrate-active enzymes (CAZymes), particularly lignin peroxidases and manganese peroxidases, which facilitated the breakdown of aromatic polymers (Rytioja et al., 2014). This enzymatic machinery not only drove litter decomposition but also promoted the formation of stable soil organic carbon (SOC) through humification processes, potentially explaining the higher SOC stocks observed in mixed stands. Empirical evidence indicated that introducing broad-leaved tree species into coniferous stands significantly enhanced Basidiomycota colonization in the rhizosphere zone, while concurrently altering fungal community structure (Li et al., 2021). These basidiomycetous fungi demonstrated remarkable phosphorus-mobilizing capacity through two synergistic mechanisms: (1) secretion of acid phosphatases and (2) production of low-molecular-weight organic acids (e.g., oxalic and citric acids) that chelated metal-phosphates (Taylor and Sinsabaugh, 2015). Consequently, the observed elevation in bioavailable phosphorus within mixed forest soils likely resulted from these microbially-mediated solubilization processes.

### 4.3 Microbial structure and function

Principal component analysis (PCA) revealed distinct clustering patterns among microbial communities, demonstrating significant structural differentiation. Bacterial communities exhibited stronger spatial separation than fungal communities across microhabitats, consistent with their higher sensitivity to microenvironmental variations (Li et al., 2023). Our data showed pronounced divergence in both bacterial and fungal community structures between rhizosphere and bulk soils. Furthermore, mixed-forest soils harbored unique microbial assemblages that significantly differed from pure stands, highlighting the dual regulatory effects of soil compartment and forest type on microbial community organization. Functional annotation of soil bacterial communities revealed six predominant metabolic guilds, though their relative abundances showed minimal variation across soil and forest types. Fungal functional groups were dominated by saprotrophic and symbiotic lifestyles across all stands. The saprotrophic fungi, as primary decomposers, drive ecosystem processes through: (1) lignocellulolytic enzyme production (e.g., laccases, lignin peroxidases) facilitating organic matter turnover, and (2) hyphal networks enhancing soil aggregation and xenobiotic degradation (Zhou et al., 2015; Sherman et al., 2014). Symbiotic fungi formed mutualistic associations characterized by: (i) extended nutrient acquisition networks, (ii) induced systemic resistance, and (iii) carbon-nutrient exchange (Xu et al., 2024; Ren et al., 2011; Wall et al., 2025). Notably, neither functional group exhibited significant abundance differences among forest or soil types, suggesting potential functional redundancy or unmeasured stabilizing factors requiring further study.

### 4.4 Network analysis

Network topological analysis revealed that microbial co-occurrence patterns reflected fundamental ecological relationships, with positive correlations suggesting potential mutualistic interactions or niche overlap, while negative correlations may indicate competitive exclusion (Faust and Raes, 2012; Ding et al., 2022). Our results demonstrated significantly higher network complexity in rhizosphere soils compared to bulk soils. Notably, mixed forest bacterial networks exhibited enhanced connectivity relative to pure conifer and broadleaf stands, supporting the hypothesis that tree species diversity promotes microbial interaction intensity. Network analysis revealed that increased topological complexity and intensified competitive interactions collectively enhanced the stability of rhizosphere microbial communities in mixed forests, as evidenced by higher robustness indices compared to pure stands (Yuan et al., 2021). Interestingly, the keystone taxa maintaining the interactions remained consistent across forest and soil types, suggesting potential functional redundancy or uncharacterized stabilizing mechanisms that warrant further investigation through targeted metatranscriptomics.

## 5 Limitations and prospects

This study had several limitations: (1) temporal variations in microbial functional dynamics were not assessed, (2) specific microbe-microbe interaction mechanisms remained uncharacterized, and (3) root exudate-mediated microbiome modulation was not quantitatively evaluated. Future research should integrate multi-omics approaches (metatranscriptomics and metabolomics) to elucidate functional gene expression patterns, implement *in situ* enzymatic activity profiling across seasons, and develop synthetic microbial communities for controlled gnotobiotic experiments to validate key drivers of observed community patterns.

## 6 Conclusion

This study confirmed that the mixed forest of *P. massoniana* and *Q. acutissima* significantly reshaped the microbial community structure by enhancing rhizosphere soil nutrients (TP, AP, AN, SOC, etc.). The key soil factors in the rhizosphere (SOC, TP, AP, AN) drived the enrichment of dominant bacterial communities (Proteobacteria, Acidobacteria) and fungal communities (Basidiomycota), and enhanced the strength of microbial network interactions. Although the changes in *α*-diversity were not significant, the unique *β*-diversity characteristics and more complex community structure of mixed forests indicated the formation of functionally specialized microbial communities. This change, by promoting nutrient cycling and enhancing ecological stability, confirmed that mixed forest transformation was an effective strategy for improving the soil ecological function of artificial forests.

## Data Availability

The data generated for this study are available on request to the corresponding author. Raw amplicon sequences were deposited in the Sequence Read Archive (SRA) and assigned the following BioProject accession number: PRJNA1270166.

## Footnotes

^1^ www.illumina.com

^2^ http://metagene.cb.k.u-tokyo.ac.jp/

^3^ http://www.ncbi.nlm.nih.gov/Taxonomy/taxonomyhome.html/index.cgi?chapter=tgencodes#SG1
