# Supplementary material for: Comparative analysis of rhizosphere microbial communities in monoculture and mixed oak–pine forests: structural and functional insights
Source: Front Microbiol. 2025 Jul 25;16:1646535. doi: 10.3389/fmicb.2025.1646535 (PMC12331662; doi:10.3389/fmicb.2025.1646535)
Supplement: Supplementary file 1 [file Data_Sheet_1.docx]

Supplementary Materials


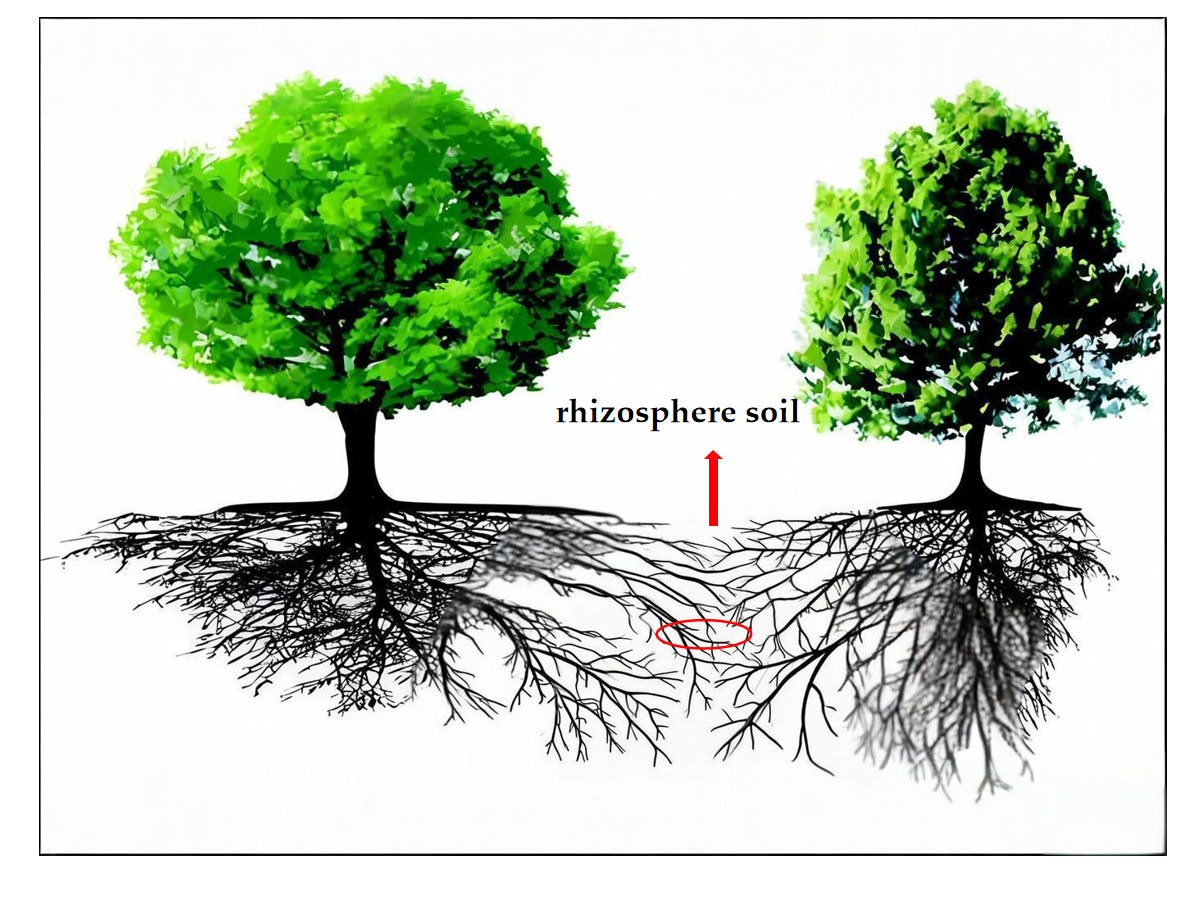


**Supplementary Figure 1**. Schematic diagram of rhizosphere soil. Non-rhizosphere soil referred to soil that lacked plant roots.
