# Supplementary material for: Comparative analysis of rhizosphere microbial communities in monoculture and mixed oak–pine forests: structural and functional insights
Source: Front Microbiol. 2025 Jul 25;16:1646535. doi: 10.3389/fmicb.2025.1646535 (PMC12331662; doi:10.3389/fmicb.2025.1646535)
Supplement: Supplementary file 2 [file Data_Sheet_2.docx]

**Supplementary Materials**

**Supplementary Table 1.** The sum of abundance of bacterial phyla (node).

| R-QA | |  | Non-QA | |  |
| --- | --- | --- | --- | --- | --- |
| node | sum abundance (%) |  | node | sum abundance (%) | n |
| Proteobacteria | 194.8 |  | Acidobacteria | 168.0 | 5 |
| Acidobacteria | 141.2 |  | Proteobacteria | 165.2 | 5 |
| Actinobacteria | 115.7 |  | Actinobacteria | 104.0 | 5 |
| Verrucomicrobia | 16.4 |  | Verrucomicrobia | 29.5 | 5 |
| Candidatus_Rokubacteria | 5.0 |  | Candidatus_Rokubacteria | 6.8 | 5 |
|  |  |  |  |  |  |
| R-PM | |  | Non-PM | |  |
| node | sum abundance (%) |  | node | sum abundance (%) | n |
| Acidobacteria | 182.7 |  | Acidobacteria | 163.1 | 5 |
| Proteobacteria | 145.6 |  | Actinobacteria | 114.1 | 5 |
| Actinobacteria | 101.5 |  | Verrucomicrobia | 14.6 | 5 |
| Verrucomicrobia | 36.7 |  | Candidatus_Eremiobacteraeota | 5.1 | 5 |
| Chloroflexi | 7.2 |  | Chloroflexi | 5.0 | 5 |
|  |  |  |  |  |  |
| R-Mix | |  | Non-Mix | |  |
| node | sum abundance (%) |  | node | sum abundance (%) | n |
| Acidobacteria | 164.6 |  | Proteobacteria | 161.8 | 5 |
| Proteobacteria | 153.6 |  | Acidobacteria | 152.7 | 5 |
| Actinobacteria | 126.3 |  | Actinobacteria | 133.1 | 5 |
| Verrucomicrobia | 21.6 |  | Chloroflexi | 7.4 | 5 |
| Chloroflexi | 8.8 |  | Candidatus_Rokubacteria | 4.1 | 5 |

Note: The top five bacterial phyla, listed in descending order of sum abundance (n=5), were shown in the table. R-QA, rhizosphere soil of *Q. acutissima*; Non-QA, non-rhizosphere soil of *Q. acutissima*; R-PM, rhizosphere soil of *P. massoniana*; Non-PM, non-rhizosphere soil of *P. massoniana*; R-Mix, rhizosphere soil in a *Q. acutissima* and *P. massoniana* mixed forest; and Non-Mix, non-rhizosphere soil in a *Q. acutissima* and *P. massoniana* mixed forest.

**Supplementary Table 2.** The sum of abundance of fungal phyla (node).

| R-QA | |  | Non-QA | |  |
| --- | --- | --- | --- | --- | --- |
| node | sum abundance (%) |  | node | sum abundance (%) | n |
| Basidiomycota | 450.0 |  | Basidiomycota | 369.3 | 5 |
| Ascomycota | 43.5 |  | Ascomycota | 97.0 | 5 |
| Mucoromycota | 5.4 |  | Mucoromycota | 26.8 | 5 |
|  |  |  |  |  |  |
| R-Mix | |  | Non-Mix | |  |
| node | sum abundance (%) |  | node | sum abundance (%) | n |
| Basidiomycota | 368.1 |  | Basidiomycota | 452.7 | 5 |
| Ascomycota | 96.9 |  | Ascomycota | 43.1 | 5 |
| Mucoromycota | 27.1 |  | Mucoromycota | 3.4 | 5 |

Note: The top three fungal phyla, listed in descending order of sum abundance (n=5), were shown in the table. R-QA, rhizosphere soil of *Q. acutissima*; Non-QA, non-rhizosphere soil of *Q. acutissima*; R-Mix, rhizosphere soil in a *Q. acutissima* and *P. massoniana* mixed forest; and Non-Mix, non-rhizosphere soil in a *Q. acutissima* and *P. massoniana* mixed forest.
